# Supplementary material for: Bimetal–Organic Framework-Loaded PVA/Chitosan Composite Hydrogel with Interfacial Antibacterial and Adhesive Hemostatic Features for Wound Dressings
Source: Polymers (Basel). 2023 Nov 9;15(22):4362. doi: 10.3390/polym15224362 (PMC10674882; doi:10.3390/polym15224362)
Supplement: Supplementary file 1 [file polymers-15-04362-s001.zip › polymers-2666992-supplementary.pdf]

Bimetal-organic framework loaded PVA/chitosan composite hydrogel with interfacial antibacterial and adhesive hemostatic features for wound dressing

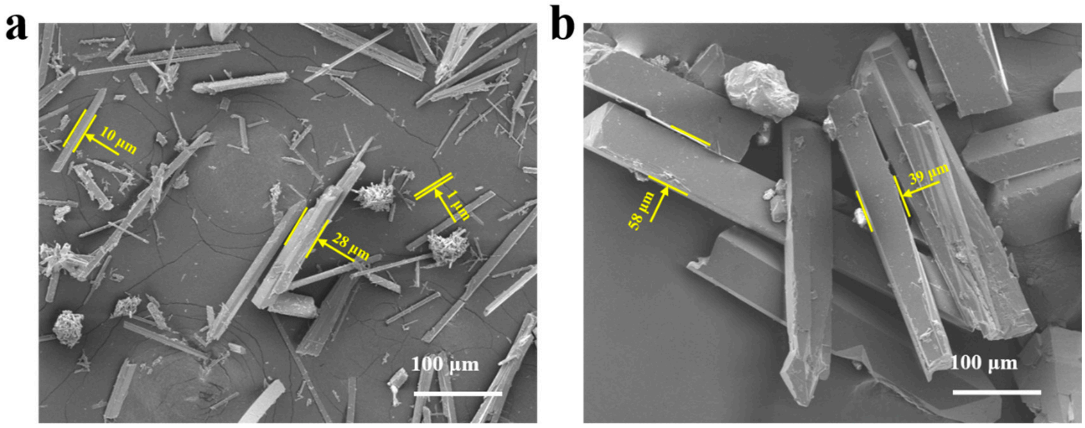

Figure S1 a) SEM image of AgMOF. b) SEM image of CuMOF.

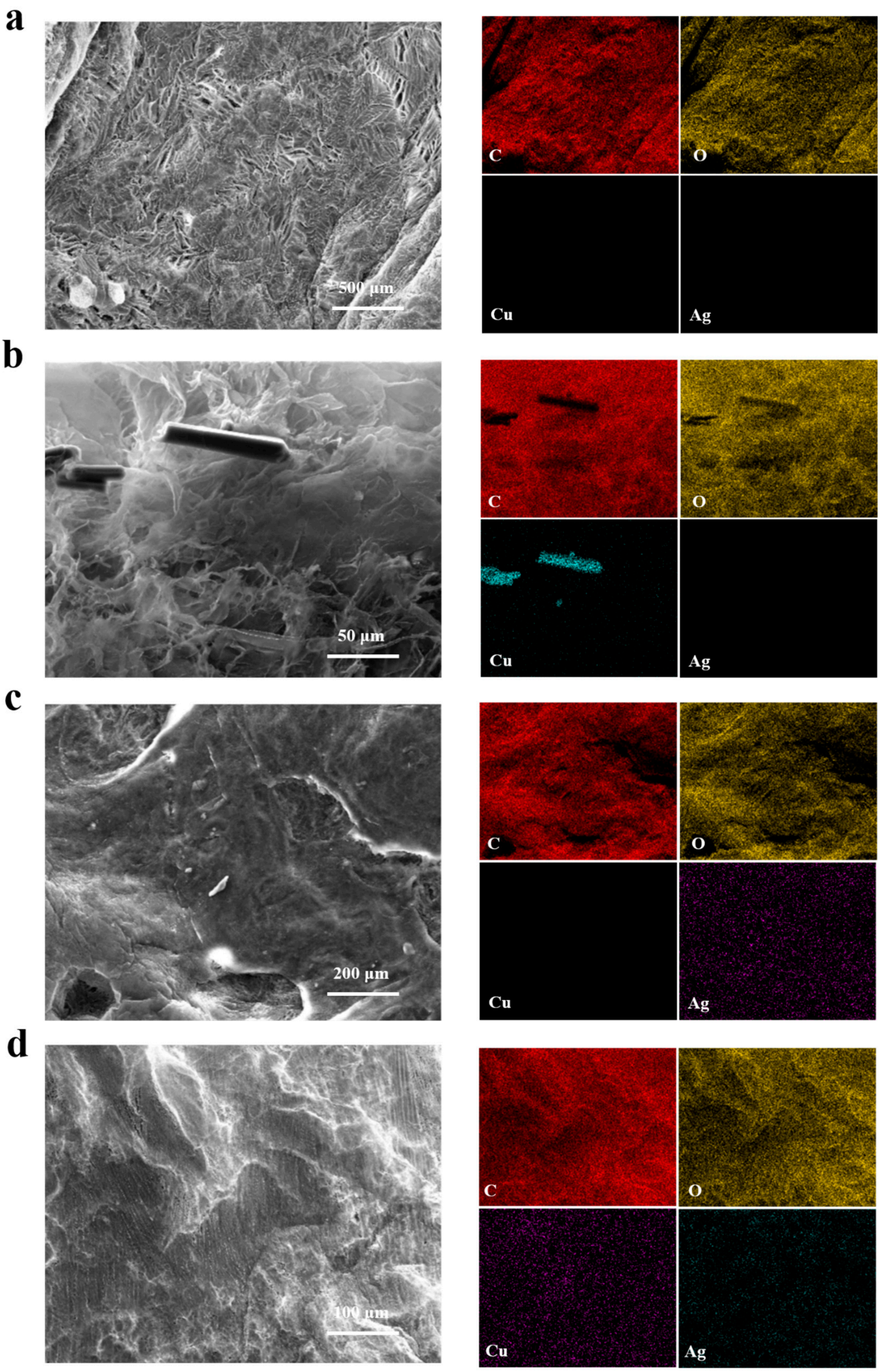

Figure S2 SEM images of a) PC, b) PCCuM, c) PCAgM, d) PCbM hydrogels and element mapping images show the distribution of C, O, Cu, Ag.

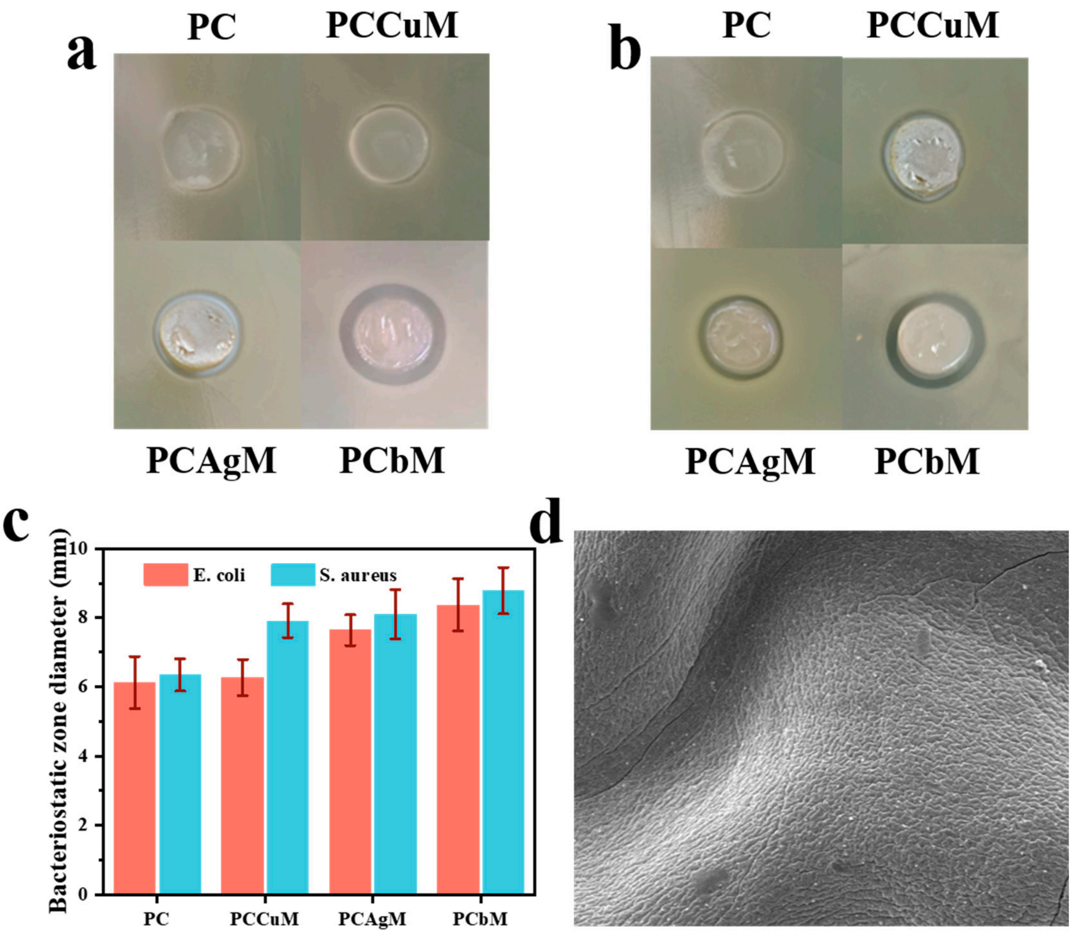

Figure S3 Inhibition zone of PC, PCCuM, PCAgM, PCbM hydrogel against a) Escherichia coli and b) Staphylococcus aureus. c) Distribution of bacteriostatic zone of PC, PCCuM, PCAgM and PCbM hydrogels. d) SEM image of the surface of PCbM hydrogel incubated in bacterial sample solution.

Measurement of metal ion concentration

The measurement method of metal ion concentration refers to the standard document *Water quality—Determination of copper—Sodium diethyldithiocabamate spectrophotometric method (HJ 485-2009)* and *Water quality—Determination of silver—Spectrophotometric method with 3,5-Br2-PADAP (HJ 489-2009)* issued by the Ministry of Environmental Protection of China. The principle of the measurement is that strong acids are used to eliminate interferences that may affect the accuracy of the experiment. A metal ion complexing agent causes the specific metal ion to develop color. Finally, the absorbance of that sample is measured by a spectrophotometer and the metal ion concentration of the sample is calculated.

Measurement of copper concentration

Take 50 ml of sample and add 5 ml of nitric acid at a concentration of 16 M to the sample and heat to eliminate. After cooling, add 5 ml of nitric acid and 1 ml of perchloric acid at a concentration of 12 M and dissolve again. After cooling, add 40 ml of water and boil for 3

min. transfer to a 50 ml volumetric flask and set the volume. Take an appropriate amount of the digested sample, put it into a dispensing funnel and add water to 50 ml. Add 10 ml of EDTA-ammonium citrate solution (5 g of disodium ethylenediaminetetraacetic acid and 20 g of ammonium citrate were dissolved in water and diluted to 100 ml, and adjusted with ammonia to pH=8-8.5. 5 ml of sodium diethyl dithiocarbamate at a concentration of 2 mg/ml was added to the mixture. Finally the organic phase was extracted with 10 ml of carbon tetrachloride). The pH was adjusted to 8-8.5 with ammonia. 5 ml of sodium diethyldithiocarbamate solution at 2 mg/ml was added and allowed to stand with shaking. Extract with 10.00 ml of carbon tetrachloride. Measure the absorbance of the organic phase at 440 nm. A calibration curve was plotted using standard concentration of copper solution. The concentration was calculated by the following equation:

$$\rho = \frac{(A - A_0) - a}{bV}$$

where ρ was the mass concentration of copper in the water sample, mg/L, A was the absorbance of the sample, A_0 was the absorbance of the reagent blank, a was the intercept of the regression equation, b was the slope of the regression equation, and V was the volume of the specimen used in the extraction, ml.

Measurement of silver concentration

Add 5 ml of nitric acid at 8 M and 1 ml of sulfuric acid at 18 M and 6.5 ml of hydrogen peroxide at 3 vt% to 25.0 ml of sample and heat to eliminate. After cooling, 2 ml of perchloric acid at a concentration of 12 M was added and heated to eliminate the solution. After cooling, 1 ml of nitric acid at 1 M concentration was added and transferred to a 25 ml volumetric flask. A suitable amount of the digested sample was placed in a volumetric flask and 1 ml of sodium citrate solution of 5 wt% was added. The solution was neutralized with 1 M sodium hydroxide solution. Sequentially add 2 ml of acetic acid-sodium acetate buffer solution (a mixture of sodium acetate at a concentration of 1 M to adjust the pH of the acetic acid solution at a concentration of 1 M to 5). Add 2 ml EDTA-2NaI at a concentration of 0.1 M, 2 ml sodium dodecyl sulfate solution at a concentration of 1 wt%, and 2 ml 3,5-Br2-PADAP at a concentration of 0.03 wt% in ethanol. The volume was fixed with deionized water. After leaving for 20 min, the absorbance at 570 nm wavelength was measured. A calibration curve was plotted with a standard concentration of silver solution. Calculations were made using the regression equation. The silver content in the sample was calculated according to the following formula:

$$\rho = \frac{m}{V}$$

Where ρ was the silver mass concentration in the water sample, mg/L, m was the silver content of the test material calculated from the calibration curve or from the regression equation, µg, and V was the volume of the sample, ml.
